# Beyond a Single Etiology: Tachy-Brady Syndrome and Possible Coexisting Focal Epilepsy Presenting as Transient Loss of Consciousness

**DOI:** 10.7759/cureus.112797

**Published:** 2026-07-16

**Authors:** Durga Naga Malleswara Rao Jonnalagadda, Deepika Beereddy, David R Murillo-Garcia, Rittu Hingorani

**Affiliations:** 1 Department of Internal Medicine, Reading Hospital - Tower Health, West Reading, USA; 2 Department of Cardiology, Reading Hospital - Tower Health, West Reading, USA

**Keywords:** focal epilepsy, sick sinus syndrome, sinus arrest, syncope, tachy-brady syndrome

## Abstract

Transient loss of consciousness has overlapping cardiac and neurologic causes, and distinguishing syncope from seizure can be difficult. The co-occurrence of a primary cardiac rhythm disorder with a possible coexisting epileptic process is infrequently reported, as most cases instead describe one condition mimicking or triggering the other. We present a case of a 77-year-old woman with hypertension, hyperlipidemia, and paroxysmal atrial fibrillation who awoke with an abnormal chest sensation, a dream-like experience, and urinary incontinence and was admitted for evaluation of a possible seizure. Continuous telemetry captured a 50-second sinus arrest (initially reported as an eight-second pause), followed by an episode of atrial fibrillation with rapid ventricular response, establishing tachy-brady syndrome secondary to severe sinus node dysfunction. The patient underwent dual-chamber pacemaker implantation. Routine electroencephalography performed the following day showed occasional left temporal focal slowing and sharp waves without captured seizures, raising concern for possible focal epilepsy; incidentally identified right-sided meningiomas were discordant with the left temporal focus. The prolonged sinus arrest occurred during sleep without accompanying clinical seizures and showed a tachy-brady pattern typical of intrinsic sinus node disease, arguing against ictal asystole. Although the telemetry findings strongly supported intrinsic sinus node dysfunction, the interictal EEG abnormalities raised the possibility of an additional epileptogenic tendency. The patient was started on levetiracetam for a provisional diagnosis of epilepsy, pending reassessment. This case illustrates that a cardiac rhythm disorder and a possible coexisting epileptic process can occur together and that identifying one etiology should not preclude investigation of the other. Clinicians should maintain a low threshold for both continuous cardiac monitoring and EEG in patients with atypical transient loss of consciousness, as findings favoring one diagnosis do not preclude the other.

## Introduction

Syncope and seizures are two common causes of transient loss of consciousness; however, distinguishing between them can be challenging because of overlapping clinical features [1]. Syncope is characterized by an abrupt, transient loss of consciousness with loss of postural tone, usually resulting from transient cerebral hypoperfusion [2]. In contrast, seizures arise from sudden, abnormal electrical activity in the brain, leading to convulsions, altered awareness, staring spells, and sometimes urinary or fecal incontinence [3,4]. Both conditions may present with similar features, such as transient unresponsiveness and urinary incontinence, making accurate diagnosis crucial, as management strategies differ significantly [5-7].

Sick sinus syndrome (SSS) is a disorder of the sinoatrial node that results in symptomatic bradycardia, sinus pauses, or alternating bradyarrhythmia and tachyarrhythmia. Tachy-brady syndrome is a form of SSS characterized by alternating bradyarrhythmia and tachyarrhythmia [8]. These disturbances reduce cardiac output and cerebral perfusion, often manifesting as syncope, presyncope, or convulsive syncope, in which transient cerebral hypoperfusion produces brief myoclonic movements that can closely resemble epileptic activity [8,9]. This overlap becomes particularly challenging when evaluating patients for focal epilepsy, as the clinical features of seizures, particularly those of temporal lobe epilepsy, can themselves mimic cardiac-related events [4]. Conversely, the neurologic symptoms arising from SSS may be misattributed to focal epilepsy, delaying appropriate treatment [9]. Confusion can also occur in the opposite direction, as seizures themselves can provoke bradyarrhythmia. In ictal asystole, seizure activity induces transient asystole through seizure-driven parasympathetic activation [10].

To our knowledge, SSS presenting alongside interictal (between seizures) EEG findings suggestive of possible focal epilepsy has rarely been reported, with the existing literature more often describing one condition mimicking or precipitating the other [10,11].

Here, we present the case of a 77-year-old woman with SSS and interictal EEG abnormalities that raised the possibility of focal epilepsy, underscoring the need to consider more than one contributing diagnosis and to pursue a comprehensive evaluation when the presentation does not fit a single etiology.

## Case presentation

A 77-year-old woman with hypertension, hyperlipidemia, and paroxysmal atrial fibrillation presented to the ED after an episode of abnormal chest sensation accompanied by urinary incontinence. At around 2:00 AM, she experienced an unusual sensation of “bubbles” in her chest upon awakening, although it was unclear whether she was fully awake or in a dream-like state. This was followed by involuntary urinary incontinence, after which she fell back asleep. The episode was unwitnessed. She denied chest pain, dyspnea, convulsions, or tongue biting. Upon awakening again at 6:00 AM, she was unsure whether the event had been real or a dream until she realized that she had been incontinent and needed to clean herself. Her medications included metoprolol tartrate 12.5 mg twice daily, lisinopril 10 mg daily, atorvastatin 10 mg nightly, and rivaroxaban 20 mg daily.

On arrival, she was hemodynamically stable, with a heart rate of 75 bpm and a blood pressure of 176/72 mmHg. Physical examination was unremarkable. Laboratory evaluation, including serial troponin measurements and a metabolic panel, was within normal limits. Electrocardiography showed normal sinus rhythm without ischemic changes. Noncontrast head CT revealed no acute intracranial pathology but incidentally identified two small meningiomas (right posterior fossa and right frontal) (Figure 1). The patient was admitted for evaluation of a possible seizure and placed on continuous telemetry. Neurology evaluated the patient, deferred antiseizure medication, and recommended EEG and MRI. Brain MRI confirmed the two extra-axial meningiomas without mass effect or acute infarction (Figure 2).

**Figure 1:**
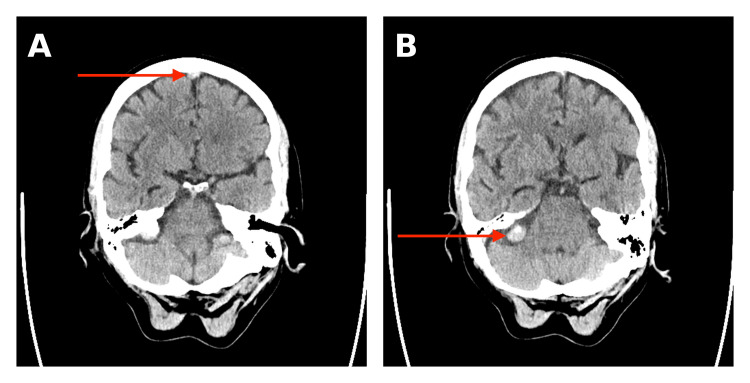
CT head without contrast. (A) A 9 × 5 mm hyperdense extra-axial lesion along the right medial frontal pole (arrow), consistent with a meningioma. (B) A 14 × 9 mm rounded hyperdense extra-axial mass along the right posterior fossa, abutting the posterior petrous ridge (arrow), also consistent with a meningioma. No midline shift or mass effect was present.

**Figure 2:**
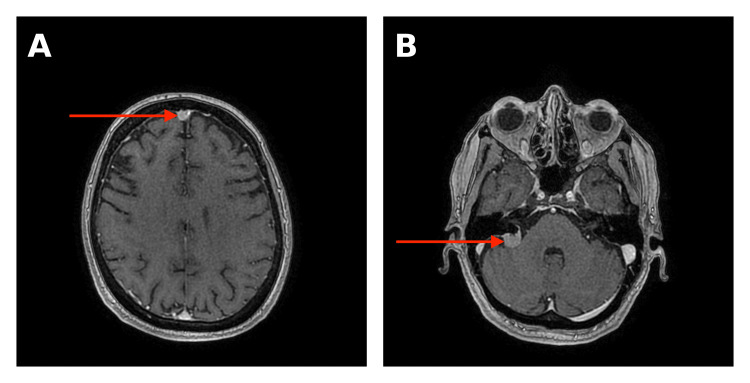
MRI brain with and without contrast confirming two extra-axial meningiomas. (A) A 7 × 7 mm enhancing extra-axial mass along the right aspect of the anterior interhemispheric fissure (arrow), consistent with a meningioma. (B) A 13 × 10 mm enhancing extra-axial mass at the right cerebellopontine angle (arrow), also consistent with a meningioma. Neither lesion showed surrounding edema, significant mass effect, or aggressive features.

On the first hospital night, the patient was asleep when continuous telemetry triggered an alarm for an eight-second sinus pause. Alerted by the central monitoring station, the bedside nurse evaluated the patient, who was arousable to sternal stimulation. Metoprolol was held, and cardiology was consulted. On review of the overnight telemetry, the pause initially reported by the central monitoring station as eight seconds was found to represent a 50-second sinus arrest, followed approximately 10 seconds later by an episode of atrial fibrillation with rapid ventricular response (Figure 3). None of these captured events were accompanied by urinary incontinence. The patient was diagnosed with tachy-brady syndrome secondary to severe sinus node dysfunction, with no reversible etiology identified. Although metoprolol can suppress sinus node automaticity, the low dose administered was insufficient to account for a 50-second arrest, implicating intrinsic sinus node dysfunction. Transthoracic echocardiography demonstrated normal left ventricular systolic function without structural abnormalities.

**Figure 3:**
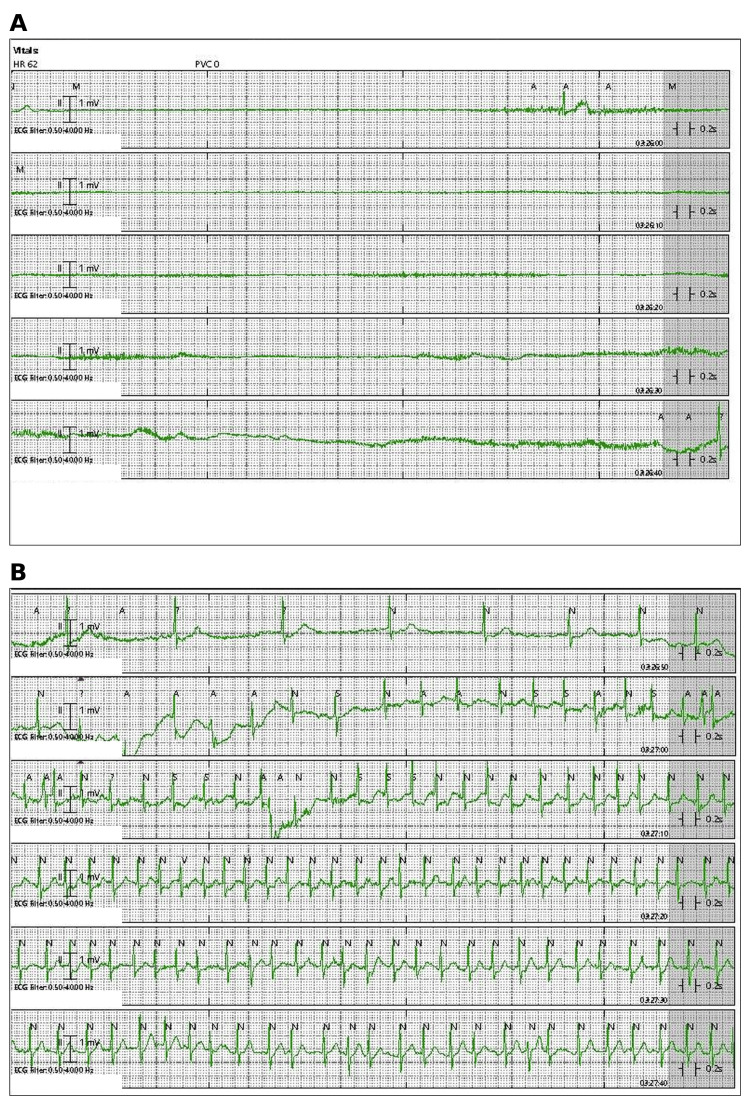
Continuous telemetry (lead II). (A) A 50-second sinus arrest occurring from 3:25:50 to 3:26:40 AM and (B) atrial fibrillation with rapid ventricular response beginning at 3:26:50 AM, consistent with tachy-brady syndrome.

On hospital day 3, a dual-chamber pacemaker was implanted without complication. Postprocedural imaging confirmed appropriate lead positioning. Routine EEG performed the following day demonstrated occasional left temporal focal slowing and sharp waves without captured seizures (Figure 4). The routine EEG captured wakefulness through drowsiness using the standard International 10-20 System of electrode placement, with one channel of ECG monitoring. No activation procedures, including photic stimulation or hyperventilation, were performed. Neurology diagnosed possible focal epilepsy. Levetiracetam was initiated with a loading dose of 1,000 mg orally once, followed by 500 mg orally twice daily.

**Figure 4:**
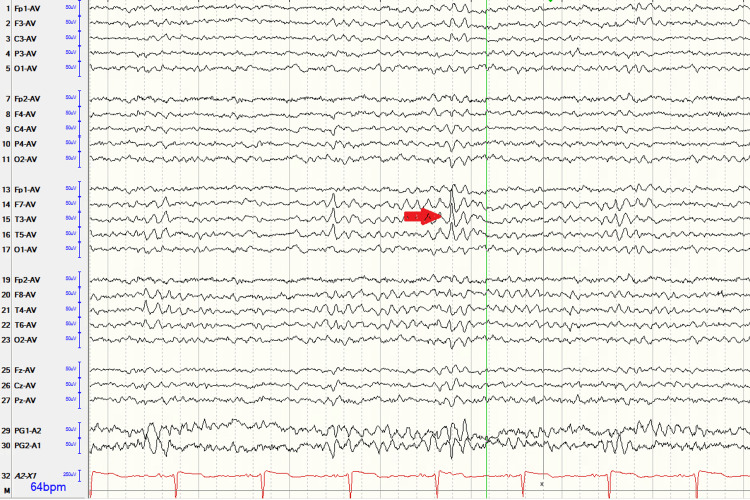
Representative EEG tracing. The EEG demonstrated occasional left temporal focal slowing and sharp waves (arrow) without captured seizures, consistent with suspected focal epilepsy.

The patient was discharged in stable condition. At the three-month outpatient follow-up, ambulatory EEG showed no epileptiform discharges. Pacemaker interrogation demonstrated atrial pacing 59.5% of the time and ventricular pacing less than 0.1% of the time, consistent with sinus node dysfunction and preserved atrioventricular conduction. The patient reported complete resolution of both the abnormal chest sensations and urinary incontinence following treatment, with no recurrence of symptoms during the follow-up period. A timeline of clinical events is presented in Table 1.

**Table 1:** Timeline of clinical events. Day 0 denotes the day of presentation to the ED; subsequent days are numbered relative to it.

Timepoint	Clinical details	Intervention
Day 0	Presented to the ED after a nocturnal episode of an abnormal bubbling chest sensation followed by urinary incontinence. CT head: incidental meningiomas. Overnight telemetry showed a prolonged sinus arrest (initially reported as eight seconds) followed by atrial fibrillation with rapid ventricular response.	Telemetry initiated; neurology consulted.
Day 1	MRI brain: two incidental meningiomas. Cardiology/electrophysiology reviewed telemetry, confirmed a 50-second sinus arrest, and diagnosed tachy-brady syndrome.	Telemetry continued. Metoprolol held due to sinus arrest; rivaroxaban held in anticipation of device implantation.
Day 3	No recurrence of symptoms; clinically stable.	Dual-chamber pacemaker implantation.
Day 4 (discharge)	Routine EEG: occasional left temporal focal slowing with sharp waves - suspected focal epilepsy (neurology).	Levetiracetam started (1000 mg loading dose, then 500 mg twice daily).
Follow-up (~3 months)	EEG normal. Pacemaker interrogation: ~60% atrial pacing with <0.1% ventricular pacing, consistent with sinus node dysfunction and preserved atrioventricular conduction.	Neurology: continue levetiracetam for two years. Cardiology: routine pacemaker follow-up.

## Discussion

The tachy-brady physiology observed in our patient is a manifestation of sinoatrial node dysfunction, which most commonly arises in elderly individuals from age-related fibrosis and degenerative changes of the conduction system [2,8]. Focal epilepsy can arise de novo in elderly patients, often in association with structural brain lesions or neurodegenerative changes [3,4,12].

Our patient’s presenting episode, an abnormal chest sensation, a dream-like experience, and urinary incontinence occurring during sleep, contained features attributable to both cardiac and neurologic etiologies. The dream-like quality and altered awareness are characteristic of temporal lobe seizures, whereas the chest sensation and urinary incontinence are recognized accompaniments of both arrhythmia-related cerebral hypoperfusion and epileptic seizures. Urinary incontinence is a poorly discriminating feature; a pooled analysis found it to be of no value in distinguishing epileptic seizures from syncope [7]. The differential diagnosis for the initial nocturnal event is broad and includes convulsive syncope resulting from the documented sinus arrest, a focal seizure with autonomic features, and, given the sleep-related onset, a parasomnia. In the absence of a witnessed account or recorded convulsive activity, these possibilities could not be reliably distinguished.

Comprehensive inpatient evaluation established tachy-brady syndrome secondary to severe sinus node dysfunction on telemetry and revealed interictal left temporal focal slowing and sharp waves on a subsequent routine EEG, raising the possibility of coexisting focal epilepsy. On this basis, the treating neurologist diagnosed possible focal epilepsy. Notably, these EEG abnormalities localized to the left temporal region, discordant with the patient’s incidentally identified meningiomas in the right frontal lobe and right posterior fossa. Their small size, extra-axial location, absence of mass effect, and lateralization opposite the EEG focus argue against the meningiomas as the epileptogenic substrate, suggesting that the focal EEG abnormality was independent of these structural lesions. No simultaneous EEG was recorded during the arrhythmic event, precluding direct correlation between the cardiac and cerebral findings. Similarly, the initial unwitnessed event at home could not be definitively attributed to either mechanism.

Ictal asystole is a rare phenomenon in which transient cardiac pauses result from seizure-driven parasympathetic activation mediated by cortical and subcortical autonomic centers [13,14]. It characteristically follows the onset of electrographic seizure activity and resolves spontaneously, with durations considerably shorter than the prolonged arrest observed in our patient [14]. Several features argue against this diagnosis: the arrest occurred during sleep without accompanying clinical seizure activity, and the tachy-brady physiology with alternating sinus arrest and atrial fibrillation is typical of intrinsic sinus node disease rather than a seizure-mediated phenomenon [8].

The diagnostic overlap between cardiac arrhythmias and epilepsy is well documented [6]. The ambiguity extends in both directions: arrhythmic episodes have been misattributed to epilepsy until an underlying conduction disorder was identified and corrected, while, conversely, cardiac arrhythmias have been directly implicated in urinary symptoms such as incontinence [15] and tachycardia-associated polyuria [16] that resolved with rhythm control. Our case adds a further dimension by raising the possibility that the two etiologies coexist, such that resolution of one symptom after treatment of one disorder does not exclude the other as a contributor to the initial presentation.

Management of this patient required a multidisciplinary approach addressing both conditions. The identification of severe sinus node dysfunction with a 50-second sinus arrest warranted permanent pacemaker implantation to prevent recurrent bradyarrhythmia-related symptoms, while the diagnosis of possible focal epilepsy necessitated initiation of levetiracetam. Because the presenting episode was interpreted as a possible first unprovoked seizure, the interictal epileptiform abnormalities were clinically meaningful, as such findings increase the risk of seizure recurrence sufficiently to support treatment after a single seizure rather than treatment of the EEG alone [17]. The concurrent use of metoprolol merited particular attention, as beta-blockers may further suppress sinus node automaticity in patients with underlying SSS. Accordingly, metoprolol was held upon discovery of the sinus arrest and reintroduced only after pacemaker implantation. Notably, an outpatient ambulatory EEG obtained approximately three months after initiation of levetiracetam showed no epileptiform discharges, and the abnormal chest sensations and urinary incontinence resolved following treatment. Neither finding cleanly identifies a single mechanism: the normal EEG may reflect the effect of levetiracetam rather than the absence of an epileptic tendency, and symptom resolution followed both pacemaker implantation and initiation of antiseizure therapy. This temporal overlap is a key limitation of the case, as it precludes attributing the subsequent symptom resolution to either intervention alone. The diagnosis of focal epilepsy remained provisional because no clinical or electrographic seizure was captured, and it rested on interictal EEG findings alone. A normal interictal EEG, particularly one recorded during treatment, does not exclude epilepsy. We continued levetiracetam because interictal EEG abnormalities suggestive of focal epilepsy carry a nontrivial risk of future seizures, with a planned reassessment after a two-year seizure-free interval to resolve this residual uncertainty.

## Conclusions

This case illustrates the diagnostic complexity that arises when tachy-brady syndrome presents with symptoms that mimic seizures and interictal EEG abnormalities suggest possible coexisting focal epilepsy. Clinicians should maintain a low threshold for both continuous telemetry and EEG in patients with atypical transient loss of consciousness, as findings favoring one diagnosis do not preclude the other.
